# Development and Validation of a Predictive Model for Metastatic Melanoma Patients Treated with Pembrolizumab Based on Automated Analysis of Whole-Body [^18^F]FDG PET/CT Imaging and Clinical Features

**DOI:** 10.3390/cancers15164083

**Published:** 2023-08-13

**Authors:** Ine Dirks, Marleen Keyaerts, Iris Dirven, Bart Neyns, Jef Vandemeulebroucke

**Affiliations:** 1 Department of Electronics and Informatics (ETRO), Vrije Universiteit Brussel (VUB), 1050 Brussels, Belgium; jef.vandemeulebroucke@vub.be; 2 IMEC, 3001 Leuven, Belgium; 3 Department of Nuclear Medicine, Universitair Ziekenhuis Brussel (UZ Brussel), Vrije Universiteit Brussel (VUB), 1050 Brussels, Belgium; marleen.keyaerts@vub.be; 4 Department of Medical Oncology, Universitair Ziekenhuis Brussel (UZ Brussel), Vrije Universiteit Brussel (VUB), 1050 Brussels, Belgium; iris.dirven@uzbrussel.be (I.D.); bart.neyns@uzbrussel.be (B.N.); 5 Department of Radiology, Universitair Ziekenhuis Brussel (UZ Brussel), Vrije Universiteit Brussel (VUB), 1050 Brussels, Belgium

**Keywords:** melanoma, survival, prognosis, whole-body, [^18^F]FDG, PET/CT

## Abstract

**Simple Summary:**

Blockade of the programmed cell death protein 1 (PD-1) receptor is an established standard-of-care treatment option that significantly improves survival of patients with advanced melanoma. While a smaller proportion of the population can derive a durable remission (even cure), most patients immediately or eventually develop disease progression. Prediction of upfront resistance to therapy as well as durable responders based on biomarkers that correlate with survival is key in selecting an optimal personalised treatment plan. Previously we reported that total metabolic tumour volume (TMTV) determined by whole-body [${}^{18}$F]FDG PET/CT is a baseline predictive biomarker that deserves further investigation. A fully automated method is proposed for feature extraction from whole-body [${}^{18}$F]FDG PET/CT. The automatically and manually derived parameters produced similar results in both the feature analysis and survival prediction. This automation can offer a fast, objective and reproducible assessment of TMTV and facilitate further exploration and validation of predictive models on larger datasets.

**Abstract:**

Background: Antibodies that inhibit the programmed cell death protein 1 (PD-1) receptor offer a significant survival benefit, potentially cure (i.e., durable disease-free survival following treatment discontinuation), a substantial proportion of patients with advanced melanoma. Most patients however fail to respond to such treatment or acquire resistance. Previously, we reported that baseline total metabolic tumour volume (TMTV) determined by whole-body [${}^{18}$F]FDG PET/CT was independently correlated with survival and able to predict the futility of treatment. Manual delineation of [${}^{18}$F]FDG-avid lesions is however labour intensive and not suitable for routine use. A predictive survival model is proposed based on automated analysis of baseline, whole-body [${}^{18}$F]FDG images. Methods: Lesions were segmented on [${}^{18}$F]FDG PET/CT using a deep-learning approach and derived features were investigated through Kaplan–Meier survival estimates with univariate logrank test and Cox regression analyses. Selected parameters were evaluated in multivariate Cox survival regressors. Results: In the development set of 69 patients, overall survival prediction based on TMTV, lactate dehydrogenase levels and presence of brain metastases achieved an area under the curve of 0.78 at one year, 0.70 at two years. No statistically significant difference was observed with respect to using manually segmented lesions. Internal validation on 31 patients yielded scores of 0.76 for one year and 0.74 for two years. Conclusions: Automatically extracted TMTV based on whole-body [${}^{18}$F]FDG PET/CT can aid in building predictive models that can support therapeutic decisions in patients treated with immune-checkpoint blockade.

## 1. Introduction

The prognosis of patients with unresectable metastatic melanoma has improved significantly thanks to the development of immune checkpoint inhibitors and BRAF/MEK targeted therapies [1,2,3,4]. Antibodies that inhibit the programmed cell death protein 1 (PD-1) receptor (e.g., pembrolizumab and nivolumab) have become an approved standard treatment option [5,6,7,8,9]. Durable responses can be achieved resulting in unprecedented survival rates at five years and beyond (e.g., in the phase III CheckMate 067 trial a 6.5-year overall survival (OS) rate of 43% was observed [10]). Notably, durable clinical benefit and remission can be obtained even following elective discontinuation of immune-checkpoint blockade [2]. While treatment with an anti-PD-1 monoclonal antibody is sufficient in a smaller proportion of patients, most patients will be in need of more active treatment options in order to obtain similar benefits. The combination of nivolumab with the cytotoxic T-lymphocyte-associated antigen 4 (CTLA-4) immune checkpoint blocking monoclonal antibody ipilimumab can improve treatment efficacy at the cost of increased toxicity [10]. Likewise, combination of the anti-PD-L1 monoclonal antibody atezolizumab with the BRAF-/MEK-inhibitors vemurafenib and cobimetinib (and the combination of spartalizumab plus dabrafenib and trametinib alike) can provide a small incremental benefit; however, this comes at the cost of increased toxicity in patients with BRAF V600-mutant melanoma [11,12].

Reliable baseline identification of patients who derive the greatest benefit from anti-PD-1 monotherapy and identification of those patients in need of more active combination therapy would be helpful to guide personalised treatment decisions. However, predicting if a specific patient will respond to anti-PD-1 therapy remains challenging and current approaches are imperfect. Some of these approaches are time-consuming and costly and have therefore not been implemented in clinical routine [13].

Translational research performed by our group and others has indicated that several parameters at baseline can predict the outcome of patients with advanced melanoma when treated with pembrolizumab [14,15,16,17]. We recently identified total metabolic tumour volume (TMTV) as determined on fluorine-18-fluorodeoxyglucose ([${}^{18}$F]FDG) positron emission tomography (PET)/computed tomography (CT) as the strongest predictor for futility of treatment with pembrolizumab [16]. In addition, in univariate analysis, a higher number of metastatic sites, the presence of brain metastases, an increase in lactate dehydrogenase (LDH) or C-reactive protein (CRP) levels, lower albumin and absolute lymphocyte count, a higher neutrophil-to-lymphocyte ratio and increased circulating tumour deoxyribonucleic acid (DNA) levels are of interest. Tissue biomarkers could not be validated, but have been shown to be of interest by others [18,19,20]. None of these biomarkers are currently widely accepted for decision making in clinical routine. With respect to determining TMTV on PET images, the extraction of the image-derived variables is too labour intensive and time-consuming and further validation is required.

Automated medical image analysis, and namely lesion segmentation, could automate the extraction of image biomarkers, alleviate the workflow and enable their usage in clinical practice. It may also allow the exploration of a wider range of image-derived parameters during clinical research studies. Recently, a large and growing body of literature has investigated lesion segmentation for PET/CT [21,22,23,24,25,26,27]. However, to the best of our knowledge, the effect of adopting such an automated approach in the pipeline for melanoma prognosis prediction remains to be investigated.

### 1.1. Related Work

A number of publications describe machine-learning methods for prognosis prediction of melanoma. Goussault et al. [28] compared a linear model, random forest, XGBoost and LightGBM to predict the response to immunotherapy and targeted therapy in stage IIIc or IV melanoma patients. A total of 935 patients from 10 different centres, taken from the French Clinical Database of Melanoma Patients (RIC-Mel), were included, of which, 80% were used for training. The response was classified as Class 1 in case of complete response, partial response or stable disease, or Class 2 in case of progressive disease. For immunotherapy, LightGBM was the best model with an accuracy of 66% while for targeted therapy, this was the random forest with an accuracy of 65%. The most predictive parameters proved to be the following: stage (IIIc or IV), response to previous treatment lines, age, number of metastasis sites and time between first melanoma excision and metastatic relapse.

Flaus et al. [29] developed a method to predict a patient’s one-year OS and progression-free survival (PFS) based on the pre-treatment [${}^{18}$F]FDG PET. The population included 56 patients treated for metastatic melanoma with anti-PD1 immunotherapy. Lesions were segmented semi-automatically using a threshold set at 40% of the maximum standardised uptake value (SUV${}_{max}$). Per patient, the lesion with the highest FDG uptake was used to extract 45 radiomic features. After a number of feature selection steps, the five best-ranked ones were used to build survival prediction models. Data were balanced through the synthetic minority oversampling technique. A neural network, logistic regression, support vector machine, random forest and naive bayes approach were compared by averaging the results of 50 random splits stratified on outcome. Each time, the training set comprised 75% of the data. For both OS and PFS, the random forest obtained superior results with an area under the curve (AUC) of 0.87, a sensitivity of 0.79 and a specificity of 0.95 for OS; and an AUC of 0.90, a sensitivity of 0.88 and a specificity of 0.91 for PFS.

Küstner et al. [30] developed a convolutional neural network (CNN) for outcome prediction and performed a range of survival analyses based on whole-body [${}^{18}$F]FDG PET/magnetic resonance imaging (MRI) and PET/CT acquired on the same day before treatment. Data from 37 patients who received checkpoint inhibitors and/or BRAF/MEK inhibitors were collected in a prospective study. A CNN was trained on automatically segmented lesions in the dataset to classify the patient as high-risk or low-risk. The latter was assigned in case of response to treatment and OS of more than 548 days. Inference was performed based on one, manually selected target lesion per patient. Results were computed in a leave-one-out cross-validation. In addition to this classification network, Kaplan–Meier analyses were performed, where patients were split into two groups and the difference in OS was assessed through a Wilcoxon test. Treatment response was evaluated with a t-test for equal means and unequal variances. The CNN for risk classification achieved a specificity of 96%, sensitivity of 92%, positive predictive value of 92% and accuracy of 95%. This model, combining all modalities, achieved superior overall results. A model based on only PET/MRI proved less sensitive and less accurate, but more specific than a model considering only PET/CT. Longer OS was seen in patients with a TMTV under 50 mL, no metastases in the brain, bone, liver, spleen or pleura, less than five affected organ regions, no metastases, a longest lesion diameter of less than 37 mm, a peak standardised uptake value of less than 1.3, or a range of mean apparent diffusion coefficient of less than 600 mm${}^{2}$. However, none of these correlated significantly with the split of patients into high- or low-risk groups.

### 1.2. Goal and Contributions of This Study

Though treatment with checkpoint inhibitors has become the new standard for advanced melanoma, a considerable part of the population still progresses on such therapy. Identification of patients that will not respond to anti-PD-(L)1 treatment at an early stage is of utmost importance to offer them the highest possible chances of survival. However, it is currently impossible to predict prior to treatment initiation if it will be effective for a specific patient. The whole-body FDG PET/CT scans that are taken before and during treatment are valuable sources of information. However, the use of quantitative image-derived parameters in clinical routine is not feasible with the available tools. There is a need for a fast, reproducible workflow to analyse the images that can be applied in clinical practice. Automation can provide the basis for this and can additionally offer tools to extract more specific imaging features that can be investigated in clinical research.

The contribution of this TRIPOD 2a [31] study is threefold. First, in addition to known imaging features, like TMTV, more specific volumetric features were extracted and assessed for their predictive power. The potential of the features was determined through univariate Kaplan–Meier and Cox regression analyses. Second, promising features were exploited and combined with clinical parameters in multivariate Cox regressions to develop a fully automated prognosis prediction model. The proposed method starts from the whole-body PET/CT image in Digital Imaging and Communications in Medicine (DICOM) format, derives all needed parameters from the metadata and preprocesses the imaging data, completes a lesion segmentation, combines extracted features with clinical parameters that are given as input and performs a prognosis prediction for patients with advanced melanoma that will be treated with pembrolizumab. The final prognosis prediction model was validated on an internal dataset from the same institute. Third, a comparative analysis was performed between manually and automatically derived imaging parameters. The impact of using the latter was evaluated in each step of the survival analysis, feature selection and prognosis prediction. In case of similar results, the automation may enable the use of quantitative image-derived parameters for therapy selection in clinical routine. Moreover, it could provide new tools and features to explore in further clinical research.

## 2. Materials and Methods

**Figure 1:**
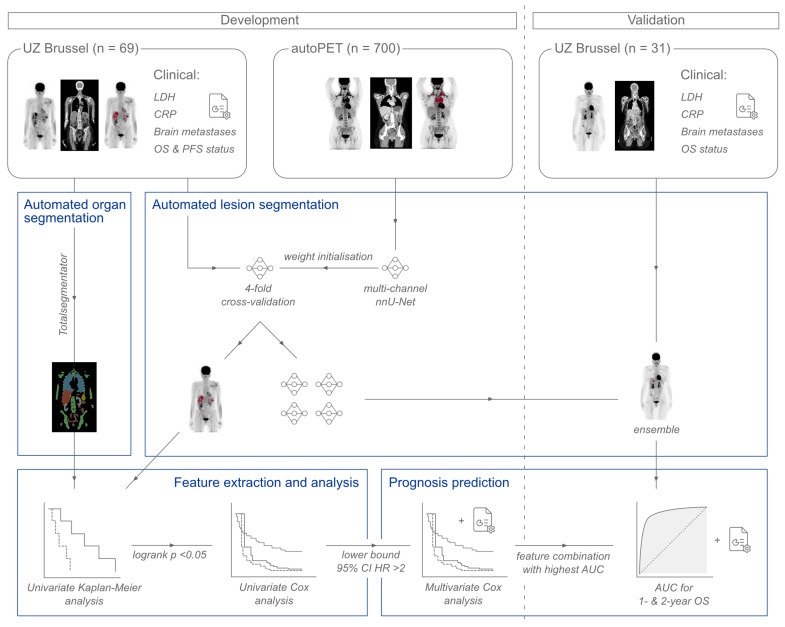
Workflow followed for the development and validation of the proposed prognostic model. In the development stage, automated segmentations were performed for the organs using Totalsegmentator [32] and for the lesions using a multi-channel nnU-Net, pretrained with data from the autoPET challenge [33], and retrained through 4-fold cross-validation within the development set. Image features were extracted using these masks and examined through univariate Kaplan–Meier and Cox regression analyses. Promising features according to logrank *p*-value and hazard ratio (HR) were then combined with available clinical parameters in multivariate Cox regressions for predicting overall survival (OS) and progression-free survival (PFS). Clinical parameters included lactate dehydrogenase (LDH), C-reactive protein (CRP) and the presence of brain metastases. The model achieving the highest area under the curve (AUC) through leave-one-out cross-validation on the development set was retrained on all patients to obtain the prognosis prediction model. In the validation stage, lesions were automatically segmented through an ensemble of the 4 models trained during development. The trained regression model was applied to the validation set to obtain a final AUC score for 1- and 2-year overall survival prediction.

This section describes the steps taken for the development and validation of the proposed prognosis prediction model. These are illustrated in Figure 1. In brief, features are derived from automatically segmented lesions and anatomical regions. Next, a feature selection is performed via univariate Kaplan–Meier and Cox regression analyses. Promising features are then evaluated in multivariate Cox regressions and the model obtaining the best results within the development set is verified on the validation set.

### 2.1. Data

This retrospective study was performed with data collected from patients treated at Universitair Ziekenhuis Brussel (UZ Brussel, Brussels, Belgium) for malignant melanoma between February 2014 and August 2018. Patients with histologically confirmed, non-resectable stage III or IV malignant melanoma according to the American Joint Committee of Cancer (AJCC) 8th edition were included. They received pembrolizumab immunotherapy every 3 weeks as 1st- or up to 5th-line treatment. Alternative prior-line therapy could consist of anti-CTLA-4 (ipilimumab), anti-PD-1 (nivolumab), a combination of anti-CTLA-4 and anti-PD-1 treatments, BRAF inhibitors (dabrafenib or encorafenib) and/or BRAF/MEK inhibitors (dabrafenib/trametinib or encorafenib/binimetinib).

A total of 100 patients were included in this study. For 69 of them, manual lesion delineations were available, created as described in [21]. This set was used for development while the remaining 31 patients were kept aside as an internal validation set.

For each patient, a whole-body [${}^{18}$F]FDG PET/CT scan was acquired at baseline, a maximum of 7 weeks before the start of pembrolizumab treatment, and at defined follow-up visits. The intervals of [${}^{18}$F]FDG PET/CT exams corresponded to roughly 3–4 immunotherapy administrations. For most patients, lesions were annotated manually by the physician as described in [21].

CRP and LDH values of a baseline blood test were recorded and categorised based on the upper limit of normal (ULN) with 5 groups for CRP (<ULN, 1–2 × ULN, 2–5 × ULN, 5–10 × ULN, >10 × ULN) and 3 groups for LDH (<ULN, 1–2 × ULN, >2 × ULN). Additionally, the presence of brain metastases at baseline was retained as a binary, categorical variable. To this end, patients suspected of having brain lesions will undergo an MRI exam a few days before or after the acquisition of the [${}^{18}$F]FDG PET/CT.

Treatment response was determined according to the immune-related response criteria (irRC) [34]. Progressive disease was defined as an increase in tumour volume of at least 25% perceived on CT. Survival was considered progression-free in case of stable disease, complete response or partial response, characterised by a reduction in tumour volume of at least 50%.

### 2.2. Automated Lesion Segmentation

The lesion segmentation model developed in a previous work [21] was adapted to overcome some of its limitations. In brief, the latter method consists of 2 steps. First, a PET threshold is automatically determined by identifying a region of interest of homogeneous intensity in the liver on both PET and CT. Application of the threshold segments all candidate regions with increased FDG uptake. In the second step, a deep learning classification is applied to separate the lesions from the healthy tissue with physiological uptake.

Here, the second step was replaced by a segmentation network using the MONAI [35] implementation of the nnU-Net [36] with 3 input channels: the binary mask from the PET thresholding, the PET and the CT image. This way, the lesion segmentation is not limited by the extent determined through thresholding, and 1 connected candidate region can be further divided into malignant and healthy tissue by the segmentation model. In accordance with the nnU-Net guidelines, a U-Net architecture [37] was trained for 1000 epochs with deep supervision, a combination of dice and cross-entropy loss and an initial learning rate of 0.01 which was decayed following ${\left(1-epoch/epoch_{max}\right)}^{0.99}$ [38]. An Adam optimizer was used instead of Stochastic Gradient Descend as this had yielded better results in previous experiments [21].

The PET intensities were converted to body-weight-corrected standardised uptake values (SUV${}_{bw}$) and clipped at 0 and 15 SUV${}_{bw}$ while CT images were clipped at −1000 and 500 Hounsfield units (HU). The intensities of both modalities were scaled to the range [0, 1] and all images were resampled to an isotropic spacing of 4 mm. Per modality, corresponding patches of 128 voxels in three dimensions were extracted while ensuring a balance between the amount of positive and negative patches. Data augmentation was performed on the fly via a number of random transformations including affine transformation, Gaussian smoothing, intensity scaling, Gaussian noise and flips, each with a probability of 0.15.

The model was first pretrained on 700 patients of the publicly available data from the autoPET challenge [33]. Since the ground truth segmentations were constructed differently, the trained model would produce inferior segmentation results for the data used in this study. Therefore, the CNN, with its weights initialised from the pretraining, was retrained on the dataset from UZ Brussel in a four-fold cross-validation. Final segmentations for the test set were constructed by averaging the output of the four models.

### 2.3. Automated Organ Segmentation

The use of automated methods for medical image analysis enables the exploration of additional, more fine-grained features for survival analysis. As an initial feasibility study, different anatomical structures were segmented using the publicly available TotalSegmentator [32]. All bones were merged into one skeleton mask. For the gastrointestinal (GI) tract, the esophagus, stomach, duodenum, small bowel, colon and urinary bladder were merged. Furthermore, the lungs, liver, spleen, adrenal glands and pancreas were located. Survival analyses were performed for the tumour load per region to investigate if there are any critical levels that could indicate treatment with pembrolizumab to be futile.

### 2.4. Feature Extraction and Analysis

Within the dataset, total metabolic tumour volume, total lesion glycolysis (TLG), baseline LDH, CRP and the presence of brain metastases were available for survival analyses corresponding to clinical research. Furthermore, tumour load in terms of TMTV per anatomical area was assessed, using the organ segmentation described in Section 2.3. All analyses were performed using Python 3.7 and packages scikit-survival and lifelines.

For the development set, each lesion-based, image-derived parameter was extracted once from the manual segmentations and once from the automated segmentations to perform univariate Kaplan–Meier analyses. For each feature, a threshold was applied to divide the population into two groups. For each group, the Kaplan–Meier survival curve was drawn and the statistical difference between both was determined through a logrank test. Different thresholds were assessed depending on the value range of the feature. The step size was set to respectively 1, 5, 10 and 100 for ranges between 1–10, 10–100, 100–1000 and more than 1000. The lowest considered threshold was equal to the step size and the maximum one was equal to the highest value under the maximum feature value. The threshold with the lowest associated *p*-value was retained for the considered feature.

The most significant of the obtained thresholds were compared across manual delineations and automated lesion segmentations to evaluate the effect of slightly different lesion tracings on the survival analysis. Hazard ratios were determined via a univariate Cox regression for overall and progression-free survival for each feature surpassing the critical threshold determined by the manual delineations and are reported with their 95% confidence interval (CI).

### 2.5. Prognosis Prediction

In order to be able to handle the right-censored data and get a prediction on a patient’s individual survival curves, the Cox proportional hazard regression was used to develop models for prognosis prediction through leave-one-out cross-validation. Hence, per experiment, each patient was used once as a test subject while training the regression on the remaining patients. The reported results are the mean values over all patients. The goal was to predict the OS and PFS chances at one year and at two years after the baseline PET/CT scan.

Volumetric features were selected based on their hazard ratio. Only features for which the lower bound of the 95% confidence interval of the hazard ratio surpassed 2, were considered in the regression modelling. The hazard ratios were calculated in a univariate, dichotomous analysis using a threshold optimised for this dataset and are therefore expected to be over-optimistic. Parameters with a hazard ratio below 2 were considered unlikely to hold information that would improve the multivariate regression models. Continuous variables were not categorised in order not to lose valuable information by thresholding.

Patients for whom TMTV rapidly drops to 0 mL are easily identified as responders through inspection of the first follow-up imaging. For the remaining patients, the prediction of response will be of interest to determine who will benefit from a continuation of the treatment. An additional Cox regression was tested to make a new survival prediction after the first follow-up PET/CT exam. For this, the rate of change in TMTV was added as a feature, and defined as

(1) $$rate\;of\;change=\frac{TMTV_{2}-TMTV_{1}}{t_{2}-t_{1}}\;,$$

with ${\mathrm{TMTV}}_{1}$ and ${\mathrm{TMTV}}_{2}$ the total metabolic tumour volume derived from the baseline and first follow-up scan, respectively, and $t_{2}-t_{1}$ the number of days between those acquisitions.

### 2.6. Evaluation

Lesion segmentations were evaluated using the dice similarity coefficient (DSC) and absolute volume difference (AVD), defined as

(2) $$DSC=\frac{2\;TP}{2\;(TP+FP+FN)}\;,$$

(3) $$AVD\;=\;|V_{ground\;truth}-V_{prediction}|\;,$$

with TP the number of true positives, FP the number of false positives and FN the number of false negatives at voxel level.

Additionally, two metrics defined in the autoPET challenge [39] were assessed, namely the volume of false positive connected components in the predicted segmentation mask that do not overlap with tumours in the ground truth segmentation map ($V_{\mathrm{FP}}$) and the volume of connected components in the ground truth segmentation that do not overlap with the estimated segmentation mask ($V_{\mathrm{FN}}$).

The overall predictive performance of regression models for survival was quantified via the AUC of the receiver operating characteristic (ROC) curve with 95% CI, determined through bootstrapping the predictions with replacement in 1000 iterations. Thus, 1000 variations of the prediction set were created by sampling these predicted probabilities and allowing each one to be sampled multiple times. The CI was then constructed based on the sampling distribution estimated from the various prediction sets. AUCs for the regression model based on manual lesion delineation versus the ones based on automated segmentations were compared with the DeLong test [40,41]. In case of multiple comparisons with the same AUC value, the Bonferroni correction was applied. We also report sensitivity or true positive rate (TPR) and specificity or true negative rate (TNR) for a threshold favouring high specificity.

(4) $$TPR=\frac{TP}{TP+FN},$$

(5) $$TNR=\frac{TN}{TN+FP},$$

with *TP* the number of true positives, *TN* the number of true negatives, *FP* the number of false positives and *FN* the number of false negatives at patient level. The 95% CI around the sensitivity and specificity was calculated by bootstrapping the predictions with replacement in 1000 iterations.

For evaluation, patients lost to follow-up were excluded from the test set as their survival status is unknown. The performance of the model was evaluated on the internal test set by retraining on all patients from the development set.

## 3. Results

### 3.1. Data

An overview of the characteristics of the development and validation subsets is provided in Table 1. The development set was made up of 69 patients (29 male, 40 female) with a median age of 60 years old (26–93). The set of whole-body [${}^{18}$F]FDG PET/CT images included at least the baseline exam, acquired a median of 7 days (0–44) before the start of the treatment, and between zero and nine follow-ups with a median follow-up time of 576 days (40–1242). A total of 16 patients had a prior history of brain metastases. The median time between PET/CT exams was 10.6 weeks (5.86–26.0). After three scans, this was increased to a median of 14.6 weeks (7.43–71.0).

One year after their baseline exam, two patients were lost to follow-up and 42 patients were still alive, of whom, 22 were progression-free. After two years, 16 patients were lost to follow-up and 22 patients survived, of whom, eight were without progressive disease. For automated lesion segmentation, the set of patients was randomly split into four groups, stratified on the number of lesions and all exams belonging to the same patient were included in the same group.

We collected an additional validation set that was never used during model development. This set comprised 31 patients (14 male, 17 female) with a median age of 65 years old (34–82) and a median follow-up time of 612 days (1–1874). Seven patients suffered from brain metastases at baseline. After one year, 18 patients were still alive while eight were lost to follow-up. A total of 13 patients survived for at least two years after their baseline exam while 10 were lost to follow-up. Manual lesion delineations and PFS status were not available for this validation set.

### 3.2. Automated Lesion Segmentation

The median segmentation results are summarised in Table 2. On average, the median dice coefficient per fold is 0.842 ± 0.343 with an absolute volume difference of 1.16 ± 239 mL. The connected components in the prediction that do not overlap with the manual delineations constitute a median volume of 0 ± 26.4 mL while the false negative ones make up 1.06 ± 35.6 mL.

### 3.3. Feature Analysis

The thresholds that led to the lowest *p*-value in a logrank test comparing Kaplan–Meier survival curves are summarised in Table 3. For each feature, the number of patients with a value higher than zero is listed as well. Univariate hazard ratios for OS and PFS determined via the manual delineations and automated lesion segmentations are tabulated in Table 4.

For OS, the hazard ratios indicated a potential predictive value in TMTV, TLG and the volume of liver, spleen and GI tract metastases at baseline. For PFS, this list was reduced to TMTV and the volume of liver metastases. Results are very similar when using manual delineations versus automatically derived lesion segmentations for TMTV, TLG and the volume of liver metastases. Hazard ratios of the volume of spleen and GI tract metastases show more variation, which can be attributed to the smaller number of patients with lesions in these areas and the more challenging nature of automated lesion segmentation in the abdomen due to the several regions of physiological uptake.

Irrespective of how the lesions were segmented, a TMTV of more than 90 mL has the highest hazard ratio for OS, followed by a tumour load in the liver greater than 30 mL and a total lesion glycolysis surpassing 400 SUV${}_{bw}\;\cdot \;$mL. For PFS, the order of liver metastases and TMTV is reversed. The most significant threshold in liver tumour load for PFS deviates with 10 mL, but there is still a significant difference (*p* < 0.001) in survival curves when applying the threshold of 30 mL instead of 40 mL in the set of automated lesion segmentations. The same can be observed for TLG. The threshold with the lowest *p*-value differs 100–200 units (for PFS and OS respectively), but the *p*-value is still smaller than 0.001 when applying the other threshold to construct the survival curves. The hazard ratio for OS based on a baseline TMTV above 90 mL is 12.2 or 14.3 when using manual delineations or automated lesion segmentations, respectively. For PFS, these values drop to 3.85 or 4.23.

A volume of liver metastases surpassing 30 mL leads to a hazard ratio of 11.0 (manual) or 8.21 (automated) for OS and 4.70 (manual) or 5.12 (automated) for PFS. A baseline TLG of more than 400 SUV${}_{bw}\;\cdot \;$mL is a bad indicator for OS with a hazard ratio of 7.77 using either method.

**Figure 2:**
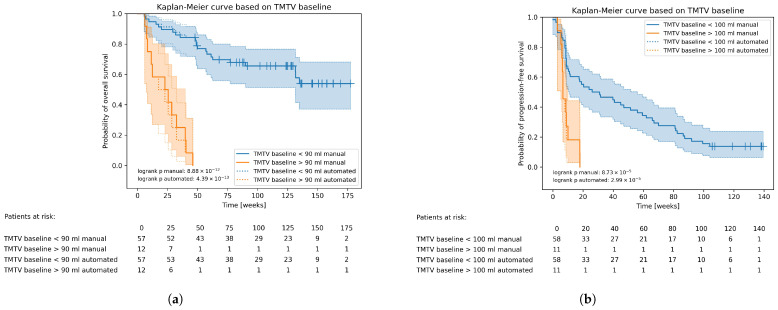
Kaplan–Meier curves and life tables based on a TMTV at baseline below or above 90 mL for OS (**a**) and below or above 100 mL for PFS (**b**).

The Kaplan–Meier curves for OS with a TMTV at baseline smaller or greater than 90 mL are drawn in Figure 2a. The plots using the manual delineations are drawn in solid lines while the ones for the automated segmentations are drawn in dotted lines. They overlap almost completely with only minor deviations at certain time points. There are two patients for which the decision of a TMTV larger or smaller than 90 mL differs, slightly modifying the plots depending on the segmentation approach. At 122 days, a patient died, for which the manual segmentation encompasses a total volume smaller than 90 mL (75.7 mL) while for the automated segmentation, this is larger than 90 mL (306 mL). The segmentation network misclassified a relatively large volume in the intestines as lesion. At 283 days, a patient died, for which the manually derived TMTV is greater than 90 mL (319 mL) while the automatically extracted value is less than 90 mL (66.2 mL), because for the latter, a large lesion in the abdomen was missed. Still, the OS curves are very similar.

The Kaplan–Meier plots for PFS based on TMTV are shown in Figure 2b and the graphs for OS and PFS based on TLG and the volume of liver metastases are added in Appendix A. The decision of baseline TLG surpassing 400 SUV${}_{bw}\;\cdot \;$mL is the same for all patients no matter the segmentation approach. Hence, their plots for OS overlap completely. The critical tumour load in the liver was found to be 30 mL, splitting the population into two groups with significantly different survival chances for both OS and PFS. The graphs only deviate slightly between segmentation methods.

In addition to the image-derived variables, CRP and LDH values and the presence of brain metastases were collected, which do not depend on the lesion segmentation method. For the blood values, the most significant threshold was determined in a similar way, examining the different categories with respect to the ULN. In line with previous research, the thresholds of 2 × ULN for LDH and 10 × ULN for CRP proved most significant [16].

A CRP level greater than 10 × ULN (*p* < 0.01) has a hazard ratio of 13.1 (95% CI: 1.53–112) for OS and 8.25 (95% CI: 0.92–73.8) for PFS.

For LDH, a value surpassing 2 × ULN (*p* < 0.001) has a hazard ratio of 13.9 (95% CI: 3.67–52.4) for OS and 7.85 (95% CI: 2.07–29.7) for PFS.

The logrank *p*-value between the groups with and without brain metastases at baseline is smaller than 0.05 with a hazard ratio of 2.33 (95% CI: 1.13–4.80) for OS while it is greater than 0.05 for PFS.

Considering this feature analysis, TMTV, TLG, the volume of liver, spleen and GI tract metastases are potential parameters to be used in a prognosis prediction model for OS. For PFS, the options are reduced to TMTV and the volume of liver metastases. For both prediction tasks, LDH, CRP and the presence of brain metastases are tested in the leave-one-out prognosis prediction as they hold information that can be complementary to the volumetric features.

### 3.4. Prognosis Prediction at Baseline

TLG is determined by multiplying TMTV with the PET intensity in standardised uptake values, so these are highly correlated (Pearson’s correlation of 0.95). Moreover, all patients with more than 30 mL of liver metastases at baseline that do not survive one or two years have a TMTV of more than 90 mL. Therefore, an initial prognosis prediction model was tested considering the remaining parameters.

Table 5 outlines the results through leave-one-out cross-validation for the Cox proportional hazard regressor considering TMTV, the LDH category and the presence of brain metastases. Table 6 summarises the estimated baseline survival and multivariate hazard ratios. For OS, the proposed automated pipeline achieved an AUC of 0.78 for prediction at one year and 0.70 at two years. For the former, the model estimated a baseline survival probability of 0.82 with multivariate hazard ratios of 1.004 for TMTV, 2.59 for LDH and 2.52 for brain metastases. At two years, the estimated baseline survival probability decreased to 0.73 with respective hazard ratios of 1.004, 1.93 and 2.58.

In case of PFS, the one-year prediction achieved an AUC of 0.61 and the baseline survival probability was estimated at 0.74 with corresponding hazard ratios of 1.004 (TMTV baseline), 1.72 (LDH baseline) and 1.69 (brain metastases at baseline). The two-year prediction reached an AUC of 0.42 with an estimated baseline survival of 0.51 and multivariate hazard ratios of 1.005 (TMTV baseline), 1.41 (LDH baseline) and 1.69 (brain metastases at baseline). Similar predicted survival probabilities and hazard ratios were found when using the manually segmented lesions. The addition of the lesion volume in the spleen or GI tract or of the CRP category did not offer any performance improvement. For all predictions, the DeLong test indicated no statistical difference (*p* > 0.05) in AUC when using the manual delineation or automated lesion segmentation approach.

TLG is highly correlated with TMTV, but might contain more information as it gives an indication of the tumour activity. However, the AUC values are similar for each of the respective survival types and time points for the prognosis model considering TMTV, LDH and brain metastases and the one taking into account TLG, LDH and brain metastases. The DeLong *p*-values are larger than 0.05 except for one-year OS with the manual segmentation where *p* is exactly 0.05 between the two models.

The volume of liver metastases proved to be predictive for survival, but the subset of patients with a TMTV greater than 90 mL completely encompassed the group of patients for which this value was higher than 30 mL. Moreover, for all prediction tasks, a drop in performance was observed when replacing the TMTV with the volume of liver lesions, which was statistically significant for OS and for PFS at one year. After Bonferroni correction, the statistical significance level is set to 0.02 due to the comparison of the AUC of the automated method with the values for the manual approach, the model including TLG instead of TMTV and the regression with the volume of liver lesions instead of TMTV. For the latter, only OS at two years and PFS at one year still give significantly different results.

### 3.5. Prognosis Prediction after First Follow-Up

The median time to the first follow-up was 62 days (28–154). The rate of change was added to the list of features and the 15 patients without a follow-up exam were excluded. Results of the leave-one-out cross-validation are shown in Table 7. One- and two-year OS were predicted with AUC scores of 0.68 and 0.66, respectively. Though this is a decrease compared to the model at baseline, the values cannot be compared directly as some patients were excluded here. When running the baseline model with the same patients excluded, the AUC values become 0.78 and 0.67. These values are still higher than for the follow-up model, but without a statistically significant difference (Delong *p*-values > 0.05).

For PFS, the model achieved an AUC of 0.53 at one year and 0.48 at two years. For the baseline model applied to the same set of patients, the scores were respectively 0.50 and 0.35 (Delong *p*-values > 0.05). When deriving the volumetric covariates from manually segmented lesions, similar values were obtained. The AUCs at one and two years were respectively 0.78 and 0.65 for OS and 0.44 and 0.32 for PFS. These are not statistically different from the scores obtained when using the automated segmentation approach (all DeLong *p*-values > 0.05).

There were three patients with an initial increase in TMTV (rate of change: 0.77 (0.55–1.78)) but who survived at least as long as their total follow-up time (median follow-up time: 1140 days (805–1242)). A total of 10 patients had no lesion on both their baseline and follow-up scans, leading to a rate of change of zero, but also survived at least as long as their total follow-up time (median follow-up time: 988 days (774–1183)). For these 13 patients, all predictions OS were correct, irrespective of the applied segmentation method.

### 3.6. Internal Validation Set

**Figure 3:**
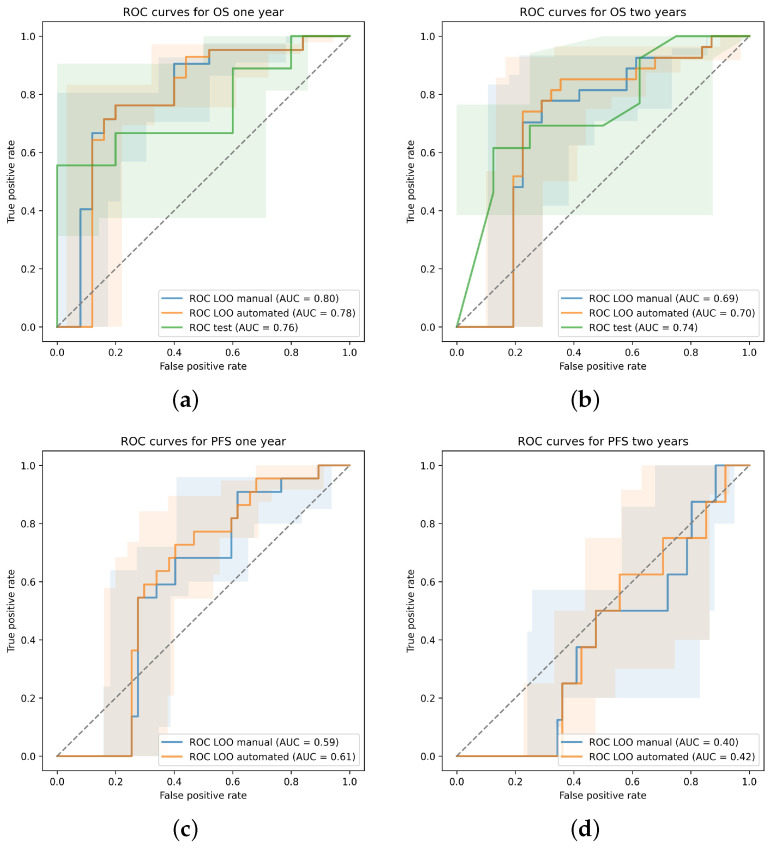
Receiver operating characteristic (ROC) curves with 95% confidence interval determined through bootstrapping for the leave-one-out cross-validation (LOO) in case of the manual and proposed automated approach and for the validation on the internal test set. Predictions are performed for (**a**) OS at one year, (**b**) OS at two years, (**c**) PFS at one year and (**d**) PFS at two years.

For the internal validation set, no PFS status was available. The multivariate Cox regression model trained on all 69 patients of the development set considering TMTV, LDH strata and brain lesions at baseline was applied to the internal test set. The ROC curves for each prediction task are drawn in Figure 3. For one-year OS, the AUC reached 0.76 (95% CI: 0.52–0.95) with a sensitivity of 0.61 (95% CI: 0.38–0.83) and specificity of 0.81 (95% CI: 0.33–1.00). At two years, the AUC became 0.74 (95% CI: 0.46–0.93) with a sensitivity of 0.61 (95% CI: 0.32–0.87) and specificity of 0.74 (95% CI: 0.40–1.00).

**Figure 4:**
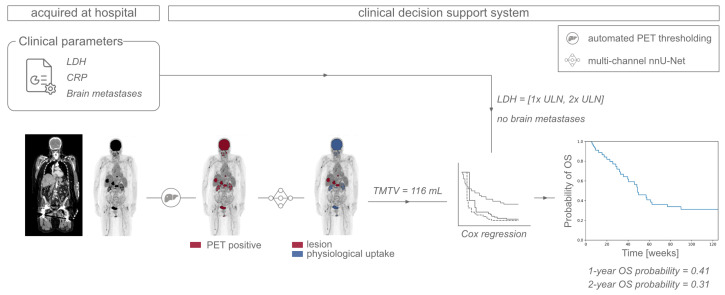
Example of the proposed clinical decision support system applied to a new patient. The PET, CT and clinical parameters are acquired at the hospital. The PET and CT are used by an automated PET threshold selection method [21] to generate a mask of all areas of increased tracer uptake. Next, the three images are given as input to a multi-channel nnU-Net [36] to segment the lesions. From this segmentation, the TMTV is extracted and combined with the LDH category and a binary parameter indicating the presence of brain metastases. Based on these variables, a multivariate Cox regression estimates the overall survival curve for the considered patient.

To illustrate the use and output of the system, the proposed clinical decision support system (CDS) is depicted in Figure 4.

## 4. Discussion

A predictive model based on fully automated analysis of whole-body [${}^{18}$F]FDG PET/CT was developed and validated on a separate dataset acquired at the same institute. Special attention was given to the impact of using automatically estimated lesion segmentations versus manual delineations.

Univariate analysis led to highly similar features being selected using automated lesion segmentation with respect to manual delineation. The prognosis prediction, including TMTV, LDH strata and the presence of brain metastases at baseline, gave results with no statistically significant difference with respect to the manual lesion delineation. This important result indicates that while the automated approach leads to slightly different lesion segmentations than obtained through manual delineations, the deviations are small and not of impact for final prognosis prediction.

Lesions in the abdomen were found to be the most challenging, bearing most discrepancies between the automated and manual segmentations. This region is characterised by higher physiological uptake, hampering the performance of the segmentation model. Segmentation models specifically trained for the abdominal region, as proposed by Jemaa et al. [42], may improve this behaviour.

Despite the good overall agreement of the automated prognostic model with respect to the manual approach, a clinical implementation of the decision support system could still allow for user interaction. A possible implementation is illustrated in Figure 4. In the automated PET thresholding step, the clinician can alter the selected threshold to change the segmentation of PET-positive regions. Next, after the lesion segmentation step, the output shows both the lesions and areas that are classified as physiological tracer uptake. If needed, the user can select delineated components to be included or excluded from the tumour load. Such an implementation would still improve the speed and reproducibility of the process, while improving the interpretability of the results.

Considering the predictive value of image-derived features, TMTV was found to lead to the best overall performance. It should be noted that the baseline [${}^{18}$F]FDG PET/CT acquisition did not always coincide with the start of the treatment. As a result, there might be an underestimation in TMTV at the start of the treatment. Future research in which all PET/CT exams are performed close to start of treatment may yield a further improvement in results.

**Figure A1:**
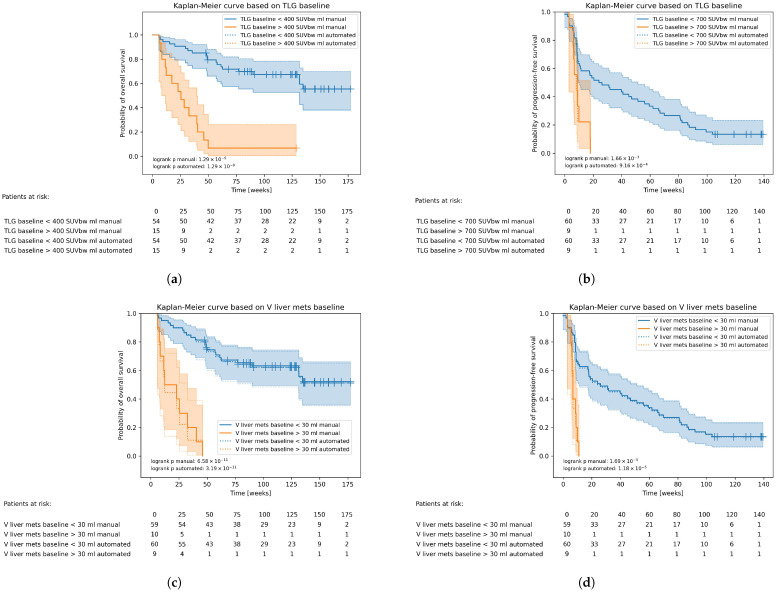
Kaplan–Meier curves and life tables based on a TLG at baseline below or above 400 SUV${}_{bw}\;\cdot \;$mL for OS (**a**) and below or above 700 SUV${}_{bw}\;\cdot \;$mL for PFS (**b**), a volume of liver metastases at baseline below or above 30 mL for OS (**c**) and for PFS (**d**).

TLG includes information on the tumour size and intensity in SUV. Therefore, theoretically, it could be preferred over TMTV as a feature. Moreover, slight oversegmentations will be less pronounced after multiplication with PET intensity. This can be observed in the perfect agreement between manual and automated segmentation of the Kaplan–Meier curves for TLG (Figure A1a). That being said, no significant difference in performance could be observed in the Cox proportional hazard prediction when including TLG instead of TMTV, and we preferred to retain TMTV for simplicity.

Both total metabolic tumour load and the liver metabolic tumour load proved highly predictive for survival. In fact, all patients with a volume of liver metastases higher than 30 mL also had a TMTV greater than 90 mL, but the opposite was not true. In case both features lead to similar performance, this would greatly reduce the workload for manual lesion delineation as only liver tumours would have to be delineated. However, for all prediction tasks, a drop in performance was observed, which was significant for several models. The volume of liver lesions cannot simply replace TMTV.

Considering the performance of the automated prognostic models, the one-year OS prediction performs well with an AUC of 0.78. One possible use of the prognosis prediction models could be to identify patients that will not respond to the standard anti-PD-1 treatment, such that an alternative treatment plan can be considered. Prioritizing sensitivity for non-responders comes at the cost of including some patients that would have responded and may be overtreated. However, such an approach could be favoured over the alternative, where patients with a poor prognosis are overlooked and do not receive the alternative therapy that might increase their chances. For automatically predicting overall survival at one year, a specificity of 0.88 was achieved with a sensitivity of 0.65, indicating that 88% of patients who would not survive one year with the standard pembrolizumab treatment can be identified correctly, at the cost of overtreating 35% of the patients. Important to note is that overtreatment by administering ineffective yet potentially toxic anti-PD-1 mono-therapy negatively impacts the overall outcome of the treated population. This highlights the importance of developing a more detailed cost–benefit analysis for deriving an optimised decision rule to determine how this prognostic model is best employed to support therapeutic decisions when starting anti-PD-1 monotherapy. In addition, patients predicted to fail anti-PD-1 monotherapy may benefit from treatment with BRAF-/MEK-inhibitors (if *BRAF* mutant) or participating in clinical trials exploring novel combination therapies.

Another possible use would be to identify the subset of patients with a baseline TMTV below 90 mL, that do not survive the first year. Both manual and automated models classify 10 out of 13 correctly. With this in mind, the proposed model can be a useful addition to the available data to support the treatment decision at baseline made in clinical practice.

At two years, the AUC score decreases to 0.70. Prognosis at a later point in time becomes more difficult as the uncertainty on the predictors increases. In addition, the more time passes by, the more patients get lost to follow-up. Out of 69 patients in the development set, only two were lost at one year, while this number increased to 11 for two years.

Predicting PFS achieved considerably lower results with AUCs of 0.61 at one year and 0.42 at two years. These predictions are generally harder due to the more specific nature of the task. The performance obtained here was not found to hold clinical value. The addition of other predictors that were not available in this dataset, might improve the estimation.

Patients whose TMTV quickly goes to 0 mL are likely to respond well to the treatment and are easy to identify at an early stage. For other cases (stable or increasing TMTV), it is harder to predict if continuation of the treatment would be beneficial. Therefore, prognosis models were tested for prediction after the first follow-up where the rate of change in TMTV was added as a feature. The overall performance of latter models in terms of AUC were not significantly different than models predicting prognosis at baseline. Considering only patients with stable or increasing TMTV at first follow-up, the model including rate of change correctly classified all patients with stable or increasing TMTV that survived at least two years.

When applying the model with baseline features to the internal validation set, never used during model development, comparable model performance was obtained, with an AUC of 0.76 for the one-year prediction and 0.74 for the two-year prediction. The results suggest our model generalises well to unseen data. We should however note that in this case the confidence intervals of the ROC curves are large due to the limited number of subjects.

Automated image analysis offers further opportunities for deriving predictive markers of response. Several authors have evaluated more advanced FDG PET/CT image-derived features in prognostic models. Küstner et al. [30] evaluated organ-specific tumour load, and found some to be predictive, though patient numbers were low. In our study, tumour loads were extracted for seven different anatomical regions. For most of them, only a small number of patients suffered from lesions in the considered area, not justifying inclusion in the prognostic model. For liver, spleen and GI tract, inclusion in the prognostic model did not improve performance. In this work, information regarding brain lesions could be included solely through a binary indicator of their presence at baseline. However, the segmentation and quantification of such metastases can enable the exploration of more specific predictors.

Despite the sexual dimorphism observed in melanoma [43], sex was not found to be a reliable predictor for prognostic outcome. In the development dataset, the difference in overall survival between the male and female groups was negligible. Within the male patients, 52% died during follow-up, while this was 48% for female patients.

The addition of radiomic features was tested, but this was deemed unfeasible due to the small dataset. Similar to the work of Flaus et al. [29], radiomic features were extracted for the lesion with the highest FDG uptake per patient, excluding patients with no lesions greater than 10 mL [44] detected on the PET/CT scan. However, this subset included only 25 patients, of which, eight survived more than one year (five progression-free) and six were still alive at two years (four progression-free). To be able to draw reliable conclusions, preference was given to omit radiomic features and perform experiments with a larger dataset, including patients with small or no baseline lesions.

The predominant limitation of this study is the relatively small datasets, both for development and validation. As a result, several strata of features were under-represented, not justifying further analysis. This included CRP and several organ-specific tumour loads. Moreover, the development set showed an overrepresentation of female patients (58%). Future work should include a more extensive validation, using an external dataset.

Furthermore, we did not have access to several parameters that have been reported to affect survival chances [16,18,19,20]. Tumour intrinsic characteristics and the immunological status of the patient are important factors influencing patient outcome. The inclusion of features like albumin and absolute lymphocyte count, neutrophil-to-lymphocyte ratio, circulating tumour DNA and protein expression on tumour cells are considered of interest for investigation as co-variables allowing even more precise prognostic prediction.

## 5. Conclusions

This study focused on the development and validation of prognostic prediction models for patients with advanced melanoma treated with pembrolizumab using a combination of automatically extracted features from the whole-body [${}^{18}$F]FDG PET/CT scan and available clinical parameters. Univariate feature analysis and multivariate survival modelling yielded very similar results when using manual delineations or automated lesion segmentations. The obtained performance for the model predicting one-year overall survival indicates it could be of benefit in clinical routine for supporting therapeutic decisions. The main limitations of the study were its relatively small dataset size and the mono-centre origin of the clinical data.

## Figures and Tables

**Table 1:** Patient characteristics.

Patient Characteristic	Development Set (*n* = 69)	Validation Set (*n* = 31)
Age (median (range))	60 (26–93)	65 (34–82)
Sex (*n* (%))		
male	29 (42.0)	14 (45.2)
female	40 (58.0)	17 (54.8)
Tumour stage (*n* (%))		
IIIB	1 (1.45)	1 (3.2)
IIIC	3 (4.35)	3 (9.7)
IIID	0 (0)	1 (3.3)
IV-M1a	11 (15.94)	7 (22.6)
IV-M1b	10 (14.49)	6 (19.4)
IV-M1c	27 (39.13)	9 (29.0)
IV-M1d	17 (24.64)	4 (12.9)
Prior treatments (*n* (%))		
0	20 (29.0)	26 (83.9)
1	37 (53.6)	4 (12.9)
2	7 (10.1)	1 (3.23)
3	4 (5.80)	0 (0)
4	1 (1.45)	0 (0)
Prior anti-CTLA-4	48 (69.6)	2 (6.45)
Prior BRAF inhibitor	5 (7.25)	0 (0)
Prior BRAF/MEK inhibitor	13 (18.8)	0 (0)
Prior anti-PD-1	1 (1.45)	1 (3.23)
Prior combination anti-PD-1 + anti-CTLA-4	0 (0)	3 (9.68)
Baseline brain lesions (*n* (%))		
yes	16 (23.2)	7 (22.6)
no	53 (76.8)	24 (77.4)
Baseline CRP (*n* (%))		
<ULN	42 (60.9)	19 (61.3)
1–2 × ULN	10 (14.5)	5 (16.1)
2–5 × ULN	6 (8.7)	3 (9.7)
5–10 × ULN	10 (14.5)	1 (3.2)
>10 × ULN	1 (1.4)	3 (9.7)
Baseline LDH (*n* (%))		
<ULN	50 (72.5)	23 (74.2)
1–2 × ULN	16 (23.2)	7 (22.6)
>2 × ULN	3 (4.3)	1 (3.2)
Follow-up		
days (median (range))	576 (40–1242)	612 (1–1874)
0 follow-up scans (*n* (%))	15 (21.7)	10 (32.3)
1 follow-up scan (*n* (%))	8 (11.6)	8 (25.8)
>1 follow-up scan (*n* (%))	46 (66.7)	13 (41.9)
Survival (*n* (%))		
OS 1 year	42 (60.9)	18 (58.1)
PFS 1 year	22 (36.2)	-
censored 1 year	2 (2.9)	8 (25.8)
OS 2 years	22 (31.9)	13 (41.9)
PFS 2 years	8 (11.6)	-
censored 2 years	16 (23.2)	10 (32.3)

*n*: number of patients, CRP: C-reactive protein, LDH: lactate dehydrogenase, ULN: upper limit of normal, OS: overall survival, PFS: progression-free survival.

**Table 2:** Median lesion segmentation results through four-fold cross-validation.

Test Fold	DSC	AVD [mL]	$V_{\mathrm{FP}}$ [mL]	$V_{\mathrm{FN}}$ [mL]
0	0.821 ± 0.350	2.02 ± 32.2	0 ± 29.0	0 ± 18.0
1	0.853 ± 0.402	0 ± 40.5	0 ± 13.2	1.41 ± 17.3
2	0.887 ± 0.291	1.22 ± 54.0	0 ± 54.8	0 ± 6.71
3	0.806 ± 0.327	1.39 ± 827	0 ± 8.63	2.84 ± 100
Average	0.842 ± 0.343	1.16 ± 239	0 ± 26.4	1.06 ± 35.6

DSC: dice similarity coefficient, AVD: absolute volume difference, $V_{FP}$: volume of false positive connected components and $V_{FN}$: volume of false negative connected components.

**Table 3:** Feature threshold values that generate the lowest logrank *p*-value when using the manual and the automated lesion segmentations within the development set. The number of patients indicates the amount of patients that have a value greater than zero for the respective feature.

Baseline Feature	*n*	Threshold Range	Threshold for OS				Threshold for PFS			
			Manual		Automated		Manual		Automated	
TMTV [mL]	45	10–450	90	*	90	*	100	*	100	*
TLG [SUV${}_{bw}\;\cdot \;$mL]	45	50–4200	400	*	600	*	700	**	600	*
V liver metastases [mL]	22	10–260	30	*	30	*	30	*	40	*
V bone metastases [mL]	19	5–75	5	**	5	*	35	***	35	***
V lung metastases [mL]	15	5–55	5	***	20	***	5	***	10	**
V metastases in GI tract [mL]	11	5–75	10	***	10	*	None		10	***
V spleen metastases [mL]	4	5–15	5	*	5	*	5	**	5	**
V metastases in adrenal glands [mL]	4	1–7	1	**	1	**	None		None	
V pancreas metastases [mL]	3	1–9	None		None		None		None	

*n*: number of patients, TMTV: total metabolic tumour volume, TLG: total lesion glycolysis, V: volume, OS: overall survival, PFS: progression-free survival, * *p* < 0.001, ** *p* < 0.01, *** *p* < 0.05.

**Table 4:** Hazard ratios for overall and progression-free survival associated with manual delineations and automatically derived lesion segmentations.

Baseline Feature	Overall Survival			Progression-Free Survival		
	Threshold	Hazard Ratio (95% CI)		Threshold	Hazard Ratio (95% CI)	
		Manual	Automated		Manual	Automated
TMTV [mL]	90	12.2 (4.95–29.8)	14.3 (5.72–35.8)	100	3.85 (1.87–7.94)	4.23 (2.04–8.77)
TLG [SUV${}_{bw}\;\cdot \;$mL]	400	7.77 (3.62–16.7)	7.77 (3.62–16.7)	700	3.18 (1.48–6.82)	3.43 (1.59–7.38)
V liver metastases [mL]	30	11.0 (4.52–26.6)	8.21 (3.46–19.5)	30	4.70 (2.17–10.2)	5.12 (2.29–11.4)
V bone metastases [mL]	5	2.81 (1.38–5.72)	3.25 (1.59–6.63)	35	3.22 (0.969–10.7)	3.22 (0.969–10.7)
V lung metastases [mL]	5	3.12 (1.07–9.08)	2.56 (1.05–6.27)	5	2.62 (1.02–6.74)	1.56 (0.706–3.45)
V metastases in GI tract [mL]	10	3.00 (1.04–8.67)	7.20 (2.63–19.7)	-	-	-
V spleen metastases [mL]	5	32.6 (2.26–470)	8.26 (1.72–39.7)	5	7.19 (0.827–62.4)	7.57 (1.65–34.8)
V metastases in adrenal glands [mL]	1	3.98 (1.36–11.6)	5.36 (1.55–18.6)	-	-	-

TMTV: total metabolic tumour volume, TLG: total lesion glycolysis, V: volume.

**Table 5:** Leave-one-out cross-validation results for a Cox proportional hazard regressor taking into account TMTV, LDH category and the presence of brain metastases.

Lesion Segmentation Method	Metric	Overall Survival		Progression-Free Survival	
		1 Year	2 Years	1 Year	2 Years
Manual	AUC (95% CI)	0.80 (0.67–0.91)	0.69 (0.54–0.83)	0.59 (0.45–0.73)	0.40 (0.22–0.60)
	Sensitivity (95% CI)	0.62 (0.47–0.78)	0.59 (0.39–0.78)	0.64 (0.43–0.84)	0.25 (0.00–0.60)
	Specificity (95% CI)	0.88 (0.75–1.00)	0.78 (0.62–0.92)	0.60 (0.45–0.75)	0.61 (0.48–0.73)
Automated	AUC (95% CI)	0.78 (0.65–0.91)	0.70 (0.56–0.85)	0.61 (0.48–0.76)	0.42 (0.25–0.60)
	Sensitivity (95% CI)	0.65 (0.50–0.79)	0.56 (0.36–0.73)	0.73 (0.54–0.91)	0 (0–0)
	Specificity (95% CI)	0.88 (0.75–1.00)	0.78 (0.62–0.92)	0.60 (0.45–0.75)	0.64 (0.52–0.76)

**Table 6:** Baseline survival probability and multivariate hazard ratios estimated a Cox proportional hazard regressor taking into account TMTV, LDH category and the presence of brain metastases.

Lesion Segmentation Method	Parameter	Overall Survival		Progression-Free Survival	
		1 Year	2 Years	1 Year	2 Years
Manual	Baseline survival chance	0.82	0.73	0.75	0.53
	HR TMTV (95% CI)	1.005 (0.00–0.01)	1.01 (0.00–0.01)	1.01 (0.00–0.01)	1.01 (0.00–0.01)
	HR LDH (95% CI)	2.64 (0.10–1.84)	1.92 (−0.16–1.47)	1.69 (−0.18–1.23)	1.34 (−0.39–0.97)
	HR brain lesions (95% CI)	2.21 (−0.05–1.64)	2.28 (0.06–1.59)	1.53 (−0.26–1.12)	1.55 (−0.19–1.07)
Automated	Baseline survival chance	0.82	0.73	0.74	0.51
	HR TMTV (95% CI)	1.004 (0.00–0.01)	1.004 (0.00–0.01)	1.004 (0.00–0.01)	1.005 (0.00–0.01)
	HR LDH (95% CI)	2.59 (0.13–1.77)	1.93 (−0.11–1.42)	1.72 (−0.12–1.21)	1.41 (−0.30–0.99)
	HR brain lesions (95% CI)	2.52 (0.07–1.78)	2.58 (0.18–1.71)	1.69 (−0.16–1.22)	1.69 (−0.11–1.15)

HR: hazard ratio, TMTV: total metabolic tumour volume, LDH: lactate dehydrogenase.

**Table 7:** Leave-one-out cross-validation results for a Cox proportional hazard regressor taking into account TMTV, LDH category, brain metastases and the rate of change in TMTV after the first follow-up.

Lesion Segmentation Method	Metric	Overall Survival		Progression-Free Survival	
		1 Year	2 Years	1 Year	2 Years
Manual	AUC (95% CI)	0.78 (0.59–0.92)	0.65 (0.45–0.84)	0.44 (0.28–0.59)	0.32 (0.15–0.52)
	Sensitivity (95% CI)	0.83 (0.71–0.93)	0.74 (0.57–0.89)	0.70 (0.50–0.90)	0.50 (0.14–0.88)
	Specificity (95% CI)	0.61 (0.33–0.88)	0.61 (0.39–0.82)	0.23 (0.11–0.39)	0.37 (0.23–0.51)
Automated	AUC (95% CI)	0.68 (0.45–0.91)	0.66 (0.45–0.86)	0.53 (0.38–0.69)	0.48 (0.26–0.71)
	Sensitivity (95% CI)	0.85 (0.74–0.95)	0.74 (0.57–0.89)	1.00 (1.00–1.00)	0.63 (0.25–1.00)
	Specificity (95% CI)	0.62 (0.35–0.90)	0.67 (0.44–0.88)	0.29 (0.15–0.45)	0.35 (0.21–0.48)

## Data Availability

The publicly available autoPET data can be accessed through [33]. The other data analysed in this study are not publicly available due to ethical/privacy reasons.

## Abbreviations

The following abbreviations are used in this manuscript:

AUC	Area under the curve
AVD	Absolute volume difference
CDS	Clinical decision support system
CI	Confidence interval
CNN	Convolutional neural network
CRP	C-reactive protein
CT	Computed tomography
DICOM	Digital Imaging and Communications in Medicine
DNA	Deoxyribonucleic acid
DSC	Dice similarity coefficient
[${}^{18}$F]FDG	Fluorine-18-fluorodeoxyglucose
GI	Gastrointestinal
HU	Hounsfield units
irRC	Immune-related response criteria
LDH	Lactate dehydrogenase
MDPI	Multidisciplinary Digital Publishing Institute
MRI	Magnetic resonance imaging
OS	Overall survival
PD	Programmed death
PFS	Progression-free survival
PET	Positron emission tomography
ROC	Receiver operating characteristic
SUV${}_{bw}$	Body-weight-corrected standardised uptake values
SUV${}_{max}$	Maximum standardised uptake value
TLG	Total lesion glycolysis
TMTV	Total metabolic tumour volume
ULN	Upper limit of normal
UZ	Universitair ziekenhuis
